# Accidental knotting and subsequent removal of a catheter from the epidural space in an adult cow: a case report

**DOI:** 10.1002/ccr3.1260

**Published:** 2017-11-07

**Authors:** Graeme M. Doodnaught, Caroline Constant, André Desrochers, Daniel S. J. Pang

**Affiliations:** ^1^ Département de Sciences Cliniques Faculté de Médecine Vétérinaire Université de Montréal Saint‐Hyacinthe Quebec Canada; ^2^ Groupe de Recherche de Pharmacologie Animale du Québec (GREPAQ) Université de Montréal Saint‐Hyacinthe Quebec Canada

**Keywords:** Analgesia, bovine, epidural catheter, extradural, knot

## Abstract

This is the first reported case in the veterinary literature of a knot in an epidural catheter. The report reviews this very rare complication and describes options for investigation and retrieval of catheters entrapped in the epidural space of any species.

## Introduction

Epidural (extradural) analgesia is a common technique in bovine medicine, providing pain relief for a wide range of procedures in conscious and anesthetized animals. Placing a catheter in the epidural space (epidural catheter) facilitates continuous and long‐term epidural analgesia by avoiding repeated needle insertions (and associated sedation and restraint) 1, 2, 3, 4.

This report describes the very rare complication of an epidural catheter looping and knotting. This complication has not previously been described in the veterinary literature.

## Case Description

An 18‐month‐old Holstein heifer (body mass 415 kg) presented to the Centre Hospitalier Universitaire Vétérinaire, Université de Montréal, with a subluxation of the right hind pastern joint. The owner described an acute episode of lameness affecting all legs a year earlier. Bilateral subluxation of the hind pasterns was observed, with resolution on the left side after several months. Based on the history, osteochondritis of the distal metacarpal and metatarsal physis was suspected.

Initial physical examination identified a 1/5 right hind lameness and slight swelling of the fetlock. Physical examination of the joints revealed dorsal subluxation of the first and second phalanges of both right hind proximal interphalangeal joints, with effusion of the digital flexor tendon sheath and contracture of the deep digital flexor tendons. No other significant findings were identified. Radiography confirmed the diagnosis. The lateromedial radiographic view showed a dorsal angulation of the joint of 55° on the affected limb, compared to 7° on the contralateral limb. There was no radiographic evidence of osteoarthritis based on the absence of irregularities, erosions, lytic lesions, or proliferation on the articular surfaces.

Arthrodesis of the proximal interphalangeal joints was proposed to correct the subluxation and written, informed consent for general anesthesia, and surgery was provided by the owner.

The heifer was fasted before surgery (no hay, grain, or water for 36, 24, and 12 h, respectively). Premedication was with xylazine (Rompun 20 mg/mL; Bayer Inc, Mississauga, ON; 0.02 mg/kg, IV) and morphine (Morphine Sulfate 10 mg/mL, Preservative‐free; Sandoz, Boucherville, QC; 0.1 mg/mL, slow IV). General anesthesia was induced with ketamine (Vetalar 100 mg/mL, Vétoquinol, Lavaltrie, QC; 2.2 mg/kg, IV) and diazepam (Diazepam 5 mg/mL; Sandoz, Boucherville, QC; 0.1 mg/kg, IV) combined in the same syringe. Orotracheal intubation (26 mm ID) was achieved by direct palpation and of the larynx with one hand while guiding the endotracheal tube between the arytenoid cartilages with the other. General anesthesia was maintained with isoflurane (Isoflurane; Fresenius Kabi, Richmond Hill, ON) carried in oxygen. Positive pressure ventilation (15 mL/kg, 8–10 breaths per minute) was initiated following connection to the anesthesia machine and ventilator (Large Animal Circuit and Ventilator, Model 2800C; Mallard Medical, Redding, CA) and adjusted to maintain normocapnia. The patient was monitored using a multiparametric anesthetic monitor (LifeWindow LW6000; Digicare Animal Health, Boynton Beach, FL; ECG, pulse oximetry, sidestream capnography, invasive blood pressure), and serial arterial blood gases were performed.

In addition to IV morphine, preoperative analgesia included flunixin (Flunazine 50 mg/mL, Vétoquinol, Lavaltrie, QC; 1.1 mg/kg, IV), a 4‐point digital block of the affected limb with bupivacaine (Sensorcaine; AstraZeneca Canada Inc., Mississauga, ON; 20 mL, 0.5%) and morphine (0.1 mg/kg) administered through an epidural catheter.

After induction, the heifer was positioned in sternal recumbency, the lumbosacral space identified by palpation, overlying hair clipped, and skin aseptically prepared. Using sterile technique, a Tuohy epidural needle (BD Tuohy Epidural Needle; BD Canada, Mississauga, ON; 18 G, 9 cm) was inserted into the epidural space with the bevel directed cranially. Correct placement was confirmed by aspiration of saline from the hub of the needle (“hanging‐drop” technique), negative aspiration, and the absence of resistance to injection. A 20‐G epidural catheter (BD Perisafe 20‐G 90‐cm Closed end Nylon catheter; BD Canada, Mississauga, ON; 90‐cm Closed end Nylon catheter) was advanced into the epidural space through the Tuohy needle. A predetermined insertion depth of 40 cm was based on the target spinal segment (first lumbar vertebrae). Following removal of the Tuohy needle, an estimated 36 cm of catheter remained within the epidural space. A filter (Epidural flat filter; Smiths Medical Canada, Markham, ON) was fitted, the catheter was confirmed patent by injecting 5 mL of saline, and 0.1 mg/kg of morphine was administered, followed by flushing the filter and catheter with saline. The catheter was sutured in place, and a sterile dressing was applied to protect the insertion site and catheter.

Following epidural catheterization, the heifer was positioned in left lateral recumbency on a padded surgical table for surgery. Arthrodesis of both proximal interphalangeal joints of the right hind limb was performed using a minimally contoured 4.5‐mm‐narrow PIP‐LCP plate (DePuy Synthes Vet, West Chester, PA) designed for equine proximal interphalangeal joint arthrodesis to achieve dynamic compression. Reduction of the subluxation was confirmed radiographically (dorsal angulation 8.5° postoperatively).

The evening following surgery (8 h after the first dose of epidural morphine), the heifer was standing and ambulating well in her box‐stall. Intravenous flunixin (1.1 mg/kg) was repeated, and another dose of morphine (0.1 mg/kg) was administered through the epidural catheter.

**Figure 1:**
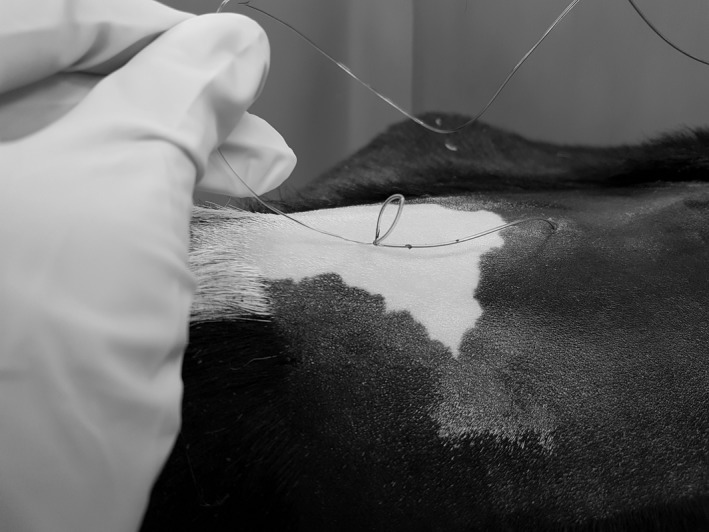
A photograph of the epidural catheter at removal, shortly after the loop and knot were exteriorized.

**Figure 2:**
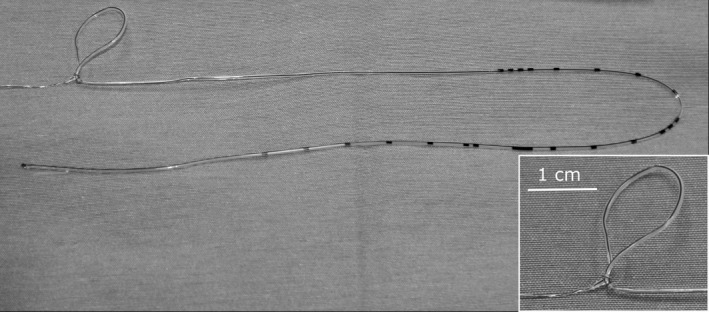
The lumbosacral epidural catheter, after removal, showing the position of the knot, centimeter gradations on catheter. (Inset) Close‐up of the knot and loop, 31 cm from the distal catheter tip.

The following day, the heifer appeared comfortable, and meloxicam (Metacam 20 mg/mL, Boehringer Ingelheim, Burlington, ON; 0.5 mg/kg, IV) was administered in the morning. Given the comfort of the patient, the decision was made to remove the epidural catheter. Without sedation, the dressing was removed, and gentle traction was used to withdraw the catheter. An estimated 4–5 cm of the catheter was exteriorized before resistance was encountered. An attempt to flush the catheter with sterile saline was unsuccessful, indicating the presence of a knot. The epidural site was aseptically prepared and, wearing sterile gloves, gentle traction was applied to the catheter. Increased resistance to withdrawal was felt as a further 1–2 cm of catheter was exteriorized. After discussing the risk of breakage and options for retrieval, the force of traction was increased, and a slight stretch in the catheter was felt just before the sensation of release. The full length of the catheter was then removed, and a knot and loop were immediately apparent (Fig. 1), 31 cm from the distal tip of the catheter (Fig. 2). No behavioral changes associated with pain were exhibited by the heifer at any point during the removal of the catheter.

The heifer remained hospitalized for 14 days following surgery, and NSAID administration (Meloxicam, 0.5 mg/kg q36 h, IV) continued for 5 days postoperatively. The heifer remained comfortable, weight bearing, and ambulating well. The day before discharge from the hospital, the cast was changed. The surgical site was healing well, and radiographs confirmed continued reduction in the subluxation.

## Discussion

To the authors' knowledge, this is the first report of knotting of an epidural catheter in the veterinary literature. Despite the description of epidural catheter placement in multiple species, including cattle 1, 2, 3, 4, 5, 6, 7, there are few prospective studies investigating the technique in animals. Knotting of epidural catheters is a very rare complication in human anesthesia, with reported incidence ranging from 1 in 20,000 to 1 in 30,000 8. Proposed contributing factors to catheter knotting include insertion depth, catheter diameter, and flexibility. The low incidence of knotting makes investigation of contributing factors difficult. Insertion depth into the epidural space is commonly perceived as a risk factor for knot formation 8, 9, 10, 11, 12, 13, 14, 15, 16, 17; however, evidence is anecdotal, based on an assumed correlation between insertion depth and knot development. Knots have been described in people with catheter insertion as little as 2–3 cm into the epidural space 18, 19, and prospective human studies evaluating catheter insertion depth of between 2 and 7 cm did not identify an association between insertion depth and knotting 20, 21.

In the reported case, the insertion distance of the catheter was selected to shorten the time to onset of analgesia by delivering morphine close to the desired site of action (approximately the third interlumbar space), as previously recommended 3, 5. A shorter insertion distance would provide effective analgesia but the onset of analgesic effect may be up to 6 h after injection 5. The low number of epidural catheter cases reported in the veterinary literature prevents estimation of the risk of knotting. In a prospective, crossover study, six bulls (24–30 months old, 335–373 kg) had catheters (16 gauge, multiport, polyamide nylon, wire reinforced) inserted approximately 48 cm into the epidural space 6. The insertion site was between the first and second coccygeal vertebrae, and catheters were advanced to the second to third lumbar space. Catheters were left in place for 4–6 weeks. At removal, knotting was not reported.

A retrospective study of the medical records of 81 dogs (5 months to 16 years old, 2.5–45.5 kg) reported catheter dislodgment as the most frequent complication (*n* = 13), followed by insertion site infection or inflammation (*n* = 2), contamination (*n* = 1), and filter breakage (*n* = 1) 4. Catheters (19 gauge, material not specified) were introduced into the epidural space at the lumbosacral junction, inserted variable distances between the seventh lumbar and fourth thoracic vertebrae, and left in place for up to 7 days (typically 2–3 days). A retrospective report of 43 equine cases had similar findings, identifying catheter dislodgement (*n* = 7), obstruction (*n* = 5), leakage (*n* = 5), and localized inflammation (*n* = 3) as complications 7. Catheter insertion depth was not reported, but insertion distances of up to 30 cm have been recommended in horses 3. In another retrospective study, the medical records of 160 dogs and 22 cats admitted to intensive care were reviewed 1. The predominant catheter type was wire reinforced, placed with and without the aid of a stylet. With a mean catheter dwell time of 50 h (range 1.3–332 h), complications observed included suspected intrathecal placement (*n* = 9), paraspinal placement (*n* = 4), pain on palpation of the lumbar region (*n* = 3), coiling of the catheter (*n* = 2), and focal necrosis of the superficial dorsal laminae with no apparent clinical signs (*n* = 1). Catheters which were difficult to place (*n* = 45) were evaluated radiographically. The author suggested that insertion depth should be limited to a few centimeters to reduce the likelihood of coiling, unless a stylet is used. In all of these studies, knotting of the catheter was not reported as a potential complication. This is the first knot observed in 47 documented epidural catheters placed in horses and cattle at the Université de Montréal over the last 11 years.

Rigid catheters (e.g., Teflon) are more easily passed through Tuohy needles and advanced into the epidural space; however, they are associated with increased frequency of intravascular cannulation, development of paresthesia (from pressure or trauma to nerve roots), inadvertent dislodgment/displacement out of the epidural space, kinking, and breakage 22. Flexible catheters are less likely to result in these complications, but are more difficult to advance and have a greater risk of coiling (and perhaps knotting). Modern catheter design aims to achieve an optimal stiffness, using polyurethane or nylon‐blend catheters with spiral wire reinforcement to impart longitudinal rigidity, with less reinforcement at the tip to increase flexibility 22. A consensus on ideal catheter material type has yet to be achieved. Polyurethane catheters as well as nylon catheters (as used in the current report) have been shown to be able to stretch more than 300% and 30% of their original length, respectively 23, 24. It is likely that the tensile strength of the catheter used in the reported case reduced the risk of breakage during retrieval.

In the current report, it appears that knot and loop formation occurred during catheter removal. This conclusion is based on being able to inject morphine solution during the first 8 h of use. In contrast, injection could not be performed during catheter removal, after resistance to extraction occurred, or after removal. Before removal, there was no indication of catheter migration, making it unlikely that a knot formed spontaneously between the last morphine injection and removal. The authors suggest that curling and/or looping of the catheter occurred as it was advanced within the epidural space. This was without consequence until removal, when traction produced cinching of the catheter and subsequent knot and loop formation. The formation of a knot and loop has been described in the human literature 13, 17, 25.

Renehan et al. and others [11, 13, 14, 15, 16, 17, 26] proposed a stepwise approach to the removal of an entrapped epidural catheter in people; (1) apply continuous gentle traction with the patient in various positions, (2) attempt to flush the catheter to determine patency (a knotted catheter is unlikely to be patent), (3) perform diagnostic imaging with the use of a guide or contrast injection to determine the position and patency of the catheter, and finally (4) explore the feasibility of surgical excision. The authors were aware of these options at the time of removal and propose this as a feasible approach to entrapped catheters in any species. Typically, gentle traction is sufficient for catheter removal, with 69% (11/16) of knotted catheters removed by this method 8, 9, 10, 11, 12, 13, 14, 15, 16, 17, 18, 19, 25, 26, 27. In the current report, when resistance to traction was encountered, the initial concern was whether the catheter had entrapped a nerve root. This appeared unlikely as traction did not cause any behavioral changes associated with pain. Had traction failed, imaging would have been attempted. The feasibility of imaging a narrow gauge catheter within the epidural space of this large patient was questioned. This option would be more practical in smaller species 4. Aseptic preparation of the insertion site was precautionary, as catheter stretching could potentially exteriorize a portion of the catheter which may have recoiled within the patient had the catheter broken. Had this occurred, remaining fragments may be of little consequence if left in place 22, 28. With this in mind, the authors' plan was to leave the catheter fragment in situ unless clinical signs warranted surgical excision.

In conclusion, the cause of epidural catheter knotting in this case is unknown. Catheter coiling within the epidural space during insertion, followed by knot and loop formation during traction at removal, is a potential explanation for the observed sequence of events. Despite this complication, the epidural catheter allowed repeated morphine delivery into the epidural space and was removed in its entirety without consequence. Further investigation is merited to determine the impact of catheter insertion depth and material properties on the incidence of knot formation and other complications in veterinary species.

## Conflict of Interest

The authors declare no conflict of interest.

## Authorship

All authors were directly involved in the clinical case described. GMD: Developed the concept and design of the report, collected clinical data, performed analysis and interpretation, drafted and revised the manuscript. CC: Analyzed and interpreted data, and revised the manuscript. AD: Supervisor (CC) and revised manuscript. DSJP: Supervisor (GMD), developed the concept and design of the report, collected clinical data, performed analysis and interpretation, drafted and revised the manuscript.
